# Benign fibroxanthoma of the mandible

**DOI:** 10.4103/0973-029X.80026

**Published:** 2011

**Authors:** L Zouloumis, C Iliopoulos, M Lazaridou, T Zarampoukas

**Affiliations:** *Associate Professor, Department of Oral and Maxillofacial Surgery, Dental School of Aristotle University of Thessaloniki, Greece*; 1 *Resident in Oral and Maxillofacial Surgery, Medical School of Aristotle University of Thessaloniki, Greece*; 2 *Associate Professor, Department of Histopathology, Medical School of Aristotle University of Thessaloniki, Greece*

**Keywords:** Benign, fibroxanthoma, histiocytoma, mandible

## Abstract

Histiocytomas constitute a large group of tumors, the classification of which created a lot of confusion in the past. For this reason, various attempts were carried out during the last years so that a widely accepted classification system could be defined. Fibroxanthomas, according to contemporary data, are classified into benign histiocytomas and they are mostly located at the skin of extremities. They are rarely localized in the area of the head and neck, where they are found commonly in soft tissue. Fibroxanthomas located at the bones of oral and maxillofacial region are extremely rare. The purpose of this paper is to present a case of a fibroxanthoma located at the mandible as well as to analyze the histological findings of the lesion on which the diagnosis and differential diagnosis were based.

## INTRODUCTION

Fibroxanthomas belong to a large group of tumors called fibrous histiocytomas which are furthermore divided into benign fibrous histiocytomas (BFHs) and malignant fibrous histiocytomas (MFHs). The term “benign fibrous histiocytoma” defines a group of well-established benign tumors commonly accompanied by hyperplasia and dive lengthening of the supernatant epithelium.

The basic histological characteristics of BFH are: uniform spindle-shaped cells and histiocyte-like tumor cells arranged in storiform pattern. Differential diagnosis of BFH generally includes ameloblastoma, myxoma, giant cell tumor, nonossifying fibroma of the childhood, gnarled peritonitis, solitary fibrous tumor, neurofibroma and leiomyoma.

## CASE REPORT

**Figure 1:**
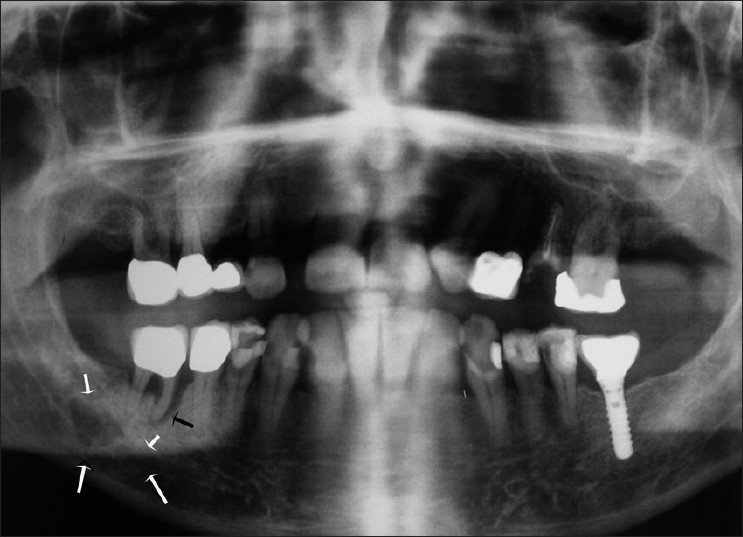
Large radiolucent area extending until the lower margin of the mandible on the right (white arrows). Vertical bone resorption toward the proximal root of 46 (black arrow)

In October 2007, a 60-year-old male arrived for assessment in our clinic with the major complaint of pain and torpor at the region of the tongue, mental nerve and infraorbital nerve. He mentioned that he had previously, for 3 weeks, taken up antibiotics without any remarkable improvement. The clinical examination involving the face and the oral cavity revealed nothing pathological concerning the mentioned symptomatology, except for tenderness in the suborbital region. The patient was soon after referred for radiological examination. The orthopantomography which was carried out revealed a 1.5 × 1 cm radiolucent lesion with rough margins located at the body of the mandible at the region distal of 46 in close relation with the mandibular canal [Figure 1].

The extraction of 46 and ectomy of the bone lesion were considered as the indicated and more appropriate therapeutic approaches. The extraction of 46 was carried out due to the non-retroverting periodontal infection (large gingival recession of the tooth with more than two-thirds of the root exposed) and serious bone absorption as demonstrated in Figure 1, which made the prognosis of the tooth very poor. Tooth extraction was followed in the same operation by the local ectomy of the bone lesion. After the elevation of a mucoperiosteal flap, the lesion was abstracted *en bloc* and the surrounding bone was debrided until health margins (bleeding bone). The procedure of debridement indeed led to the perforation of the lingual mandibular plate. The abstracted lesion was sent for histopathologic examination and antibiotics were administered to the patient for 10 days.

**Figure 2:**
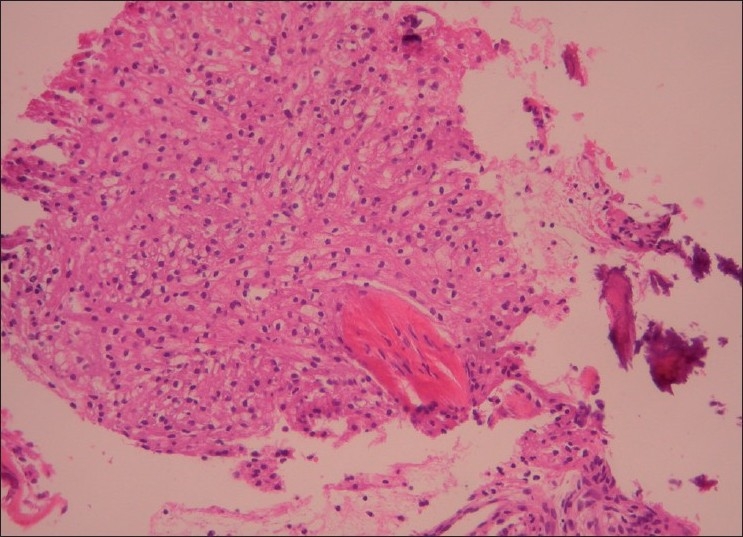
Area of a great accumulation of foamy histiocytes

**Figure 3:**
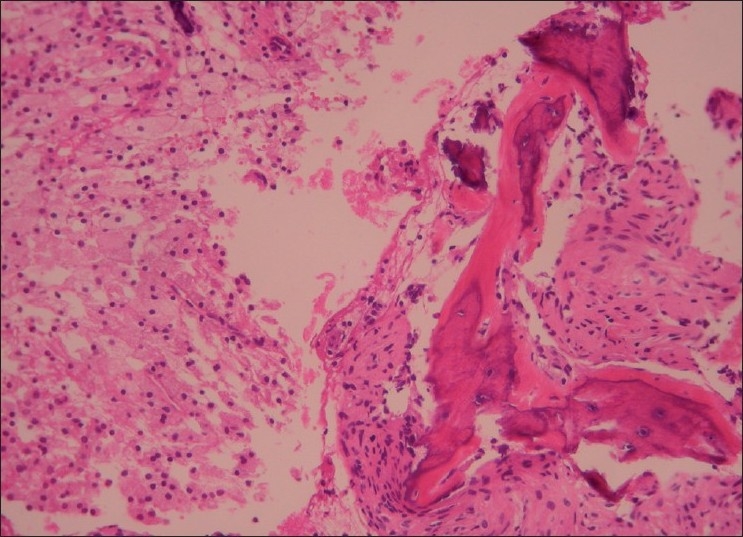
Spindle cells and many foamy histiocytes

**Figure 4:**
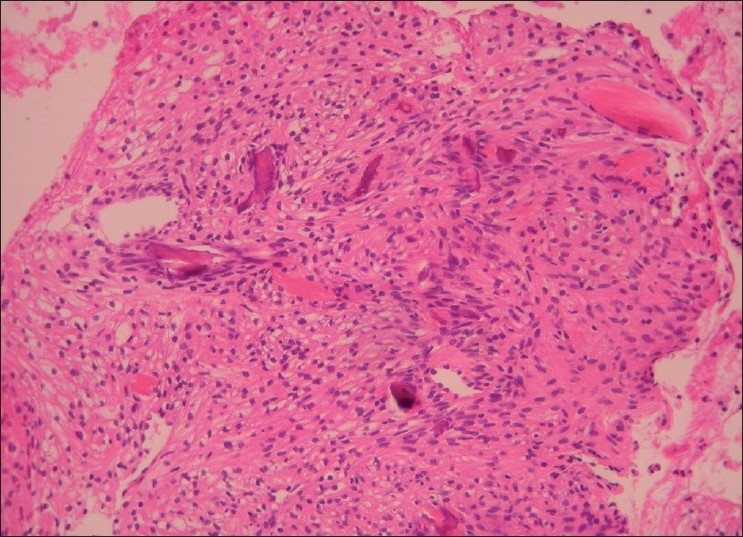
Spindle cells surrounding fragments of bone trabeculae

The histopathologic report, which arrived in our clinic 10 days later, concluded findings compatible with benign fibroxanthoma. The macroscopical findings reported included 13 brown and yellow-brown friable fragments of tissue, with 2–5 mm as the greatest diameter. Microscopically, many foamy histiocytes in dense arrangement and fewer spindle-shaped cells in fascicular and storiform arrangement were observed [Figures 2 and 3]. Among these cells, small fragments of bone trabeculae were found [Figure 4]. Malignancy was not observed.

**Figure 5:**
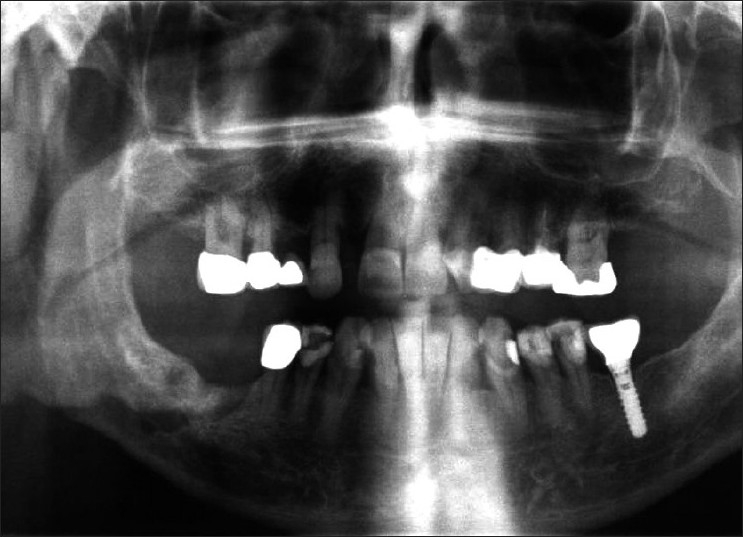
Orthopantomography 6 months postoperatively. The defect at the area of the surgical site as well as the post-extraction socket of 46 are distinguished. Nothing remarkable concerning the bone healing process is observed

The patient was recalled 15 days postoperatively, and he reported relief from the preexisting pain in face and mouth and improvement of tongue torpor, but had the complaint of remaining torpor in suborbital and mental nerve area. No signs of morbidity were noticed at the surgical site. A follow-up examination was scheduled 6 months later. At that time, both clinical and radiological examinations were performed. Clinically, the area of the surgical trauma had healed well and the orthopantomography [Figure 5] showed no signs of pathology at the site of postoperative defect.

## DISCUSSION

The term “benign fibrous histiocytoma” was first described by Stout and Lates.[1] BFHs occur both in soft tissue and bone, with the majority of them affecting the skin of extremities in the early to middle adult life.[2,3] Moreover, BFH occurs in bones such as femur, tibia and ilium.[4] Regarding its localization in oral and maxillofacial region, there have been a few reports in the literature.[5–10] Most of the reports dealt with soft tissue BFH, especially with buccal mucosa, whereas bony BFH is extremely rare. There are reports considering that the growth of these tumors is trauma or inflammation induced.[6,11] To our knowledge, there are one report of BFH in the maxilla[5] and five reports of BFH in the mandible.[12–16]

During the past, researchers had to cope with lots of difficulties in their efforts made for the constitution of a regular classification system of fibrous histiocytomas due to the limited data concerning their pathogenesis and the differentiation between BFH and MFH. Until 1960, a lot of pathological lesions had been described under the general term “fibrous histiocytoma”, with MFH not being a distinguished pathological entity. MFH is a rare mesenchymal tumor including a group of lesions such as malignant fibrous xanthoma, malignant giant cell tumor, fibroxanthoma, fibroxanthosarcoma, xanthosarcoma, inflammatory fibrous histiocytoma and malignant histiocytoma.[17,18] In 1981, Hoffman and Martinez[19] classified fibrous histiocytoma in two large groups: the benign tumors and the malignant tumors. According to them, benign tumors include the fibrous histiocytoma and the atypical fibrous histiocytoma, whereas malignant tumors include the inflammatory and the malignant fibrous histiocytomas. A more simple classification was proposed in 1992 by Gray *et al*.,[6] according to which fibrous histiocytomas are divided into BFH, atypical fibrous histiocytomas and MFH.

MFHs are tumors composed of fibroblasts and histiocytes.[20] Their origin is questionable because although it has been reported that they originate from bone, their incidence as soft tissue tumors is far more than that as bone tumors.[21] The histological characteristics of MFH include cell atypia, hyperchromatic nuclei, pleomorphism, poor cell differentiation, high mitotic activity and necrotic areas. They are located commonly at the extremities, their recurrence is common and they metastasize early locally or distantly, commonly to the lungs.[22] Due to their aggressive behavior, the recommended treatment includes early radical surgery and close follow-up. The role of adjuvant radiotherapy and chemotherapy still remains unspecified.[23]

Atypical fibroxanthoma is regarded as a superficial, less aggressive counterpart of MFH. It arises superficially and is usually confined to the dermis of the actinically damaged skin of the head and neck of the elderly.[24] Histologically, an anaplastic activity with marked cytologic atypia, increased number of mitotic figures and presence of multinucleated giant cells belies its usually benign clinical course and excellent prognosis.[25] The treatment of choice of atypical fibroxanthoma is wide surgical excision, since the possibility of recurrence is highly correlated to positive tumor margins.[18]

As it was previously mentioned, the differential diagnosis of fibroxanthoma (and BFH, in general) includes ameloblastoma, myxoma, giant cell tumor, nonossifying fibroma of the childhood, gnarled peritonitis, solitary fibrous tumor, neurofibroma and leiomyoma. In our case, the tumor had to be differentially diagnosed from ameloblastoma, odontogenic myxoma, giant cell tumor and nonossifying fibroma of the childhood. For this purpose, both clinical and microscopic characteristics of the tumor had to be assessed.

Specifically, in our case, the tumor included thin septa instead of the thick ones which are identified in ameloblastomas and are responsible for the typical soap-bubble microscopical appearance of the ameloblastoma. Furthermore, ameloblastomas, due to their destructive behavior, provoke root resorption which in our patient did not occur.

The differential diagnosis from the odontogenic myxoma was based primarily on two points: fibroxanthoma has no such destructive nature as odontogenic myxoma, and in addition, the latter one microscopically usually demonstrates straight or elongated septa.

The age of our patient and the presence of symptomatology easily excluded the possibility of a giant cell tumor of childhood.

The presence of giant cells identified in some cases of BFH causes difficulty regarding its differentiation from giant cell tumor. In our lesion, the absence of giant cell tumor as well as the spindle-shaped cells formed in storiform pattern facilitated the diagnosis.

Immunohistochemical tests including CD-68 and vimentin are also occasionally performed to state the presence of histiocytes. In our case, this was not necessary due to the immediate identification of foamy cells (histiocytes) with microscopy.

Concerning the radiological appearance of fibroxanthoma, it could not be utilized for diagnosis and differential diagnosis. This is because of the few literature reports available describing its radiographic findings.[26,27] According to these reports, some characteristics generally identified included the sclerotic rim around the lesion, low density in computed tomography (CT) and medium signal intensity at T1-weighted magnetic resonance imaging (MRI) and high signal intensity at T2-weighted MRI. According to these findings, we preferred not to carry out further radiological investigation in addition to the orthopantomography performed initially.

The suggested therapy of BFH is surgical excision. The prognosis of the lesion is excellent and there is no report that it metastasizes locally or distantly.
